# Biomarkers associated with blinatumomab outcomes in acute lymphoblastic leukemia

**DOI:** 10.1038/s41375-020-01089-x

**Published:** 2021-02-04

**Authors:** Andrew H. Wei, Josep-Maria Ribera, Richard A. Larson, David Ritchie, Armin Ghobadi, Yuqi Chen, Abraham Anderson, Cedric E. Dos Santos, Janet Franklin, Hagop Kantarjian

**Affiliations:** 1 Alfred Hospital and Monash University, Melbourne, VIC Australia; 2 ICO-Hospital Germans Trias i Pujol and Jose Carreras Research Institute, Badalona, Spain; 3 University of Chicago Comprehensive Cancer Center, Chicago, IL USA; 4 Clinical Haematology, The Royal Melbourne Hospital and Peter MacCallum Cancer Centre, Melbourne, VIC Australia; 5 Washington University School of Medicine, St. Louis, MO USA; 6 Amgen Inc., Thousand Oaks, CA USA; 7 Amgen Inc., South San Francisco, CA USA; 8 The University of Texas MD Anderson Cancer Center, Houston, TX USA

**Keywords:** Acute lymphocytic leukaemia, Drug development

## Abstract

This study aimed to identify biomarkers for clinical outcomes in a phase 3 clinical study of blinatumomab or chemotherapy in adults with Philadelphia chromosome-negative relapsed/refractory B-cell precursor acute lymphoblastic leukemia. Patients were randomized 2:1 to receive blinatumomab, a BiTE^®^ therapy, for 4 weeks (9 μg/day cycle 1 week 1, 28 μg/day thereafter) every 6 weeks, or chemotherapy. Baseline blood samples were evaluated to identify biomarkers prognostic (both treatment groups) or predictive (either treatment groups) for overall survival, event-free survival, hematologic remission, minimal residual disease (MRD) response, duration of response, or adverse events. Baseline values were balanced between treatment groups. Prognostic biomarkers were platelets, tumor burden, and percentage of T cells: each 1-log increase in platelets at baseline was prognostic for improved 6-month survival; lower tumor burden was prognostic for hematologic remission; and a higher percentage of CD3^+^ T-cells was prognostic for MRD response. Consistent with the BiTE mechanism of action, higher percentage of CD45^+^ CD3^+^ CD8^+^ T cells was associated with hematologic remission following blinatumomab. No examined biomarkers were significant for the risk of grade ≥3 adverse events. Incorporating baseline biomarkers into future studies may help to identify subgroups most likely to benefit from blinatumomab.

## Introduction

Patients with B-cell precursor acute lymphoblastic leukemia (BCP-ALL) that relapses or is refractory to conventional chemotherapy have a poor prognosis, with expected 1-year survival rate of only 26% after first salvage chemotherapy and poorer survival outcomes associated with subsequent salvage [1]. The pan-B cell marker CD19 is expressed in most (>90%) B-lineage ALL cells, making it an ideal immunotherapy target for ALL [2, 3]. Blinatumomab is a BiTE^®^ (bispecific T-cell engager) molecule that simultaneously engages CD19^+^ B cells and CD3^+^ cytotoxic T cells and redirects T cells to lyse malignant and normal B cells [4, 5].

In the randomized, phase 3 TOWER study (NCT02013167) in adults with advanced Ph^–^ relapsed or refractory BCP-ALL, blinatumomab was compared to standard-of-care chemotherapy [6]. Patients in the blinatumomab group had significantly longer overall survival, compared with those in the chemotherapy group (median, 7.7 vs. 4.0 months; *P* = 0.01), as well as a significantly higher rate of hematologic remission—CR, CRh, or CR with incomplete hematologic recovery (CRi) (44% vs. 25%; *P* < 0.001).

Hallmarks of the BiTE^®^ immuno-oncology platform, including T-cell activation, T-cell margination, redistribution, proliferation, and transient cytokine release, have been extensively described and represent pharmacodynamic characteristics of blinatumomab [7–14]. Given the increased emphasis on personalized medicine in oncology, it is becoming more important to find genetic or cellular biomarkers to identify patients who will derive the greatest benefit from immunotherapy, including blinatumomab. The objective of this post-hoc analysis was to identify baseline laboratory and immunologic biomarkers in the TOWER study that may be prognostic for clinical outcome, and to identify factors associated with clinical outcomes in the blinatumomab vs. chemotherapy group.

## Materials and methods

### Study design

The study design was described in detail in the primary report [6]. This prospective, randomized, phase 3, active-controlled study enrolled patients who were 18 years of age or older with Ph^–^ BCP-ALL that was refractory to primary induction or salvage with intensive combination chemotherapy, in first relapse with hematologic remission lasting less than 12 months, in second or greater relapse, or in relapse at any time after allogeneic stem cell transplantation. Patients were required to have more than 5% blasts in the bone marrow and an Eastern Cooperative Oncology Group performance status of 2 or less. All patients provided written informed consent. The study protocol was conducted in accordance with the Declaration of Helsinki and approved by the investigational review board or independent ethics committee at each study center. This study was registered at ClinicalTrials.gov (Identifier: NCT02013167). Amgen funded this study. Qualified researchers may request data from Amgen clinical studies. Complete details are available at http://www.amgen.com/datasharing.

Patients were randomly assigned to receive open-label treatment with either blinatumomab or standard chemotherapy in a 2:1 ratio. Randomization was stratified by age (<35 vs. ≥35 years), previous salvage therapy, and previous allogeneic stem cell transplantation. After two cycles of induction therapy, patients in hematologic remission (≤5% bone marrow blasts) could receive up to three cycles of consolidation therapy and up to 12 months of maintenance therapy. At the discretion of the investigator, protocol-specified therapy could be discontinued at any time after the first treatment cycle and the patient could subsequently undergo stem cell transplantation.

Blinatumomab induction and consolidation therapy were administered in 6 weeks cycles: 28 µg per day by continuous intravenous infusion for 4 weeks (with a lower dose of 9 μg per day during week 1 of induction cycle 1 only) and no treatment for 2 weeks. Blinatumomab maintenance was given as a 4-week continuous infusion every 12 weeks. All patients in the blinatumomab group received dexamethasone to prevent cytokine release syndrome: (1) patients with a high tumor burden during screening received dexamethasone 10 mg/m^2^/day (up to 24 mg/day) orally or by intravenous infusion for up to 21 days before the start of treatment; (2) patients without high tumor burden at screening received dexamethasone 20 mg by intravenous infusion within 1 h before each cycle of blinatumomab, before a dose increase, or after treatment interruption for >4 h; (3) all patients received intrathecal prophylaxis for central nervous system disease. Interruption or discontinuation of the dose of blinatumomab was required if neurologic events or other selected adverse events occurred. Dexamethasone was administered (orally or intravenously) to patients with cytokine release syndrome (maximum of 3 × 8 mg/day for up to 3 days, then tapered over 4 days) or a neurologic event (maximum of 24 mg/day, then tapered over 4 days).

Patients in the chemotherapy group received the investigator’s choice of 1 of 4 regimens: fludarabine, high-dose cytosine arabinoside, and granulocyte colony-stimulating factor (FLAG) with or without anthracycline; a high-dose cytosine arabinoside-based regimen; a high-dose methotrexate-based regimen; or a clofarabine-based regimen. Dose adjustment was permitted for patients receiving standard chemotherapy but was not required for specific events.

### Assessments

Complete remission was defined as 5% or lower bone marrow blasts and no other evidence of disease, with the following peripheral blood counts: CR, platelets >100,000/µL and absolute neutrophil count (ANC) > 1000/µL; CRh, platelets > 50,000/µL and ANC > 500/µL; and CRi, platelets > 100,000/µL or ANC > 1000/µL. A central laboratory for study centers in the United States and Canada assessed minimal residual disease (MRD) with multicolor flow cytometry and a central laboratory for other study centers assessed MRD with real-time quantitative polymerase chain reaction of clonal immunoglobulin or T-cell receptor gene rearrangements; assay sensitivity for MRD was at least 10^–4^ [15, 16]. Investigators graded adverse events according to the National Cancer Institute Common Terminology Criteria for Adverse Events, version 4.0.

### Baseline biomarkers

Bone marrow samples were analyzed for percentage of bone marrow lymphoblasts. Blood samples at screening were evaluated for the presence of the following baseline biomarkers, based on clinical and biological relevance: leukocytes, monocytes, neutrophils, lymphocytes, granulocytes, platelets, CD45^+^ CD3^+^ T cells absolute counts and percentage, CD45^+^ CD3^+^ CD4^+^ T cells absolute counts and percentage, CD45^+^ CD3^+^ CD8^+^ T cells absolute counts and percentage, CD45^+^ CD3^–^ CD19^+^ B cells absolute counts and percentage, ratios of CD3^+^:CD19^+^ cells, and CD3^–^ CD16^+^ CD56^+^ natural killer cells absolute counts and percentage. Peripheral blood flow cytometry studies were gated on the entire CD45^+^ population, and then gated on the side scatter defined lymphocyte gate. The percentage of lymphocyte subsets were calculated by multiplying the total lymphocyte count with the percentage of cells with the designated lymphoid phenotype (Table 1). Circulating cytokine levels were not measured.

**Table 1:** Baseline characteristics.

	Blinatumomab (*N* = 271)	Chemotherapy (*N* = 134)
Age, years, median (range)	37 (18–80)	37 (18–78)
<35, *n* (%)	123 (45.4)	60 (44.8)
≥35, *n* (%)	148 (54.6)	74 (55.2)
Male, *n* (%)	162 (59.8)	77 (57.5)
Primary refractory, *n* (%)	46 (17.0)	27 (20.1)
Prior salvage therapy, *n* (%)	164 (60.5)	80 (59.7)
Prior alloSCT, *n* (%)	94 (34.7)	46 (34.3)
Baseline cell counts, *n*; median (Q1, Q3)		
Leukocytes (10^9^/L)	271; 3.0 (1, 6)	134; 3.5 (2, 7)
Monocytes (10^9^/L)	264; 0.1 (0.0, 0.3)	131; 0.1 (0.0, 0.3)
Neutrophils (10^9^/L)	232; 1.2 (0.4, 2.7)	109; 1.5 (0.4, 3.0)
Lymphocytes (10^9^/L)	266; 0.8 (0.4, 1.4)	131; 0.8 (0.4, 1.8)
Granulocytes (/µL)	245; 1091 (383, 2 612)	85; 1157 (365, 2 893)
Platelets (10^9^/L)	271; 49 (23, 103)	134; 52 (24, 133)
CD3^+^ T cells (%)	245; 64 (33, 82)	85; 56.5 (23, 81)
CD3^+^ T cells (/µL)	245; 461 (253, 821)	85; 459 (268, 793)
CD45^+^ CD3^+^ CD4^+^ T cells (%)	245; 24.5 (11, 38)	85; 22 (10, 37)
CD45^+^ CD3^+^ CD4^+^ T cells (/µL)	245; 187 (88, 359)	85; 197 (100, 345)
CD45^+^ CD3^+^ CD8^+^ T cells (%)	245; 27 (14, 41)	85; 22 (13, 37)
CD45^+^ CD3^+^ CD8^+^ T cells (/µL)	245; 218 (109, 421)	85; 225 (105, 436)
CD45^+^ CD3^–^ CD19^+^ B cells (%)	245; 16 (2, 46)	83; 24 (4, 45)
CD45^+^ CD3^–^ CD19^+^ B cells (/µL)	245; 113 (7, 586)	83; 123 (21, 747)
CD3^–^ CD16^+^ CD56^+^ cells (%)	245; 7 (3, 14)	83; 7 (3, 10)
CD3^–^ CD16^+^ CD56^+^ cells (/µL)	245; 69 (29, 142)	83; 69 (28, 155)
CD3^+^ :CD19^+^ (E/T) ratio cells in blood	214; 2.4 (0.5, 14)	75; 1.7 (0.5, 13)
% Bone marrow blasts	243; 80 (37, 93)	129; 80 (43, 93)

Percentage of lymphocytes were calculated by dividing absolute lymphocyte subset over total lymphocyte count.

*alloSCT* allogeneic stem cell transplantation, *Q* quartile, *E/T ratio* effector-to-target ratio.

### Statistical analysis

In the screening phase, baseline biomarkers described above were first explored graphically and analyzed by univariate model. In the univariate analysis, if the main effect or treatment interaction between the biomarker and treatment had a *P* value of 0.3 or lower, these selected biomarkers entered the multivariate model to be analyzed for association with treatment outcomes. Both percentages and counts for lymphocyte subsets were analyzed. A stepwise variable selection was performed and an effect on outcome was determined to stay in the final multivariate model if either the main effect or the interaction term between the biomarker and treatment had a *P* value of 0.15 or lower. All biomarkers were treated as continuous variables in the analysis. Prognostic and predictive associations of baseline biomarkers with hematologic remission or MRD response were assessed by multivariate logistic regression. Prognostic and predictive associations of baseline biomarkers with overall survival, event-free survival, and duration of response were assessed by multivariate Cox regression. Prognostic biomarkers were those that defined the effects of patient or tumor characteristics on patient outcome in both treatment groups; predictive biomarkers were those that predicted patient outcome differently between treatment groups. Predictive biomarkers were identified using interaction tests with treatment group. Biomarkers were considered to be prognostic (both groups) or predictive (one group) if the 95% confidence interval (CI) for the odds ratio (OR; hematologic remission; MRD response) or the hazard ratio (HR; overall survival) did not include 1. The effects of baseline biomarkers on toxicity were determined by univariate logistic regression.

## Results

### Baseline characteristics

Patients were enrolled from January 2014 through September 2015. A total of 405 patients received blinatumomab (*n* = 271) or chemotherapy (*n* = 134). Demographics, baseline disease characteristics, and baseline cell counts were generally balanced between the treatment groups (Table 1). Median age was 37 years in each treatment group; overall, 59% of patients were male. Many patients were heavily pretreated for BCP-ALL before enrollment; 41% were treated in first salvage and 59% in second or later salvage. The proportion of patients treated with post-allograft relapse was 35%. Median bone marrow blasts were 80% in each treatment group. The median ratio for CD3^+^:CD19^+^ (T:B lymphocytes) was 2.4 (range, 0.5–14) in the blinatumomab group and 1.7 (range, 0.5–13) in the chemotherapy group.

### Overall survival

**Fig. 1:**
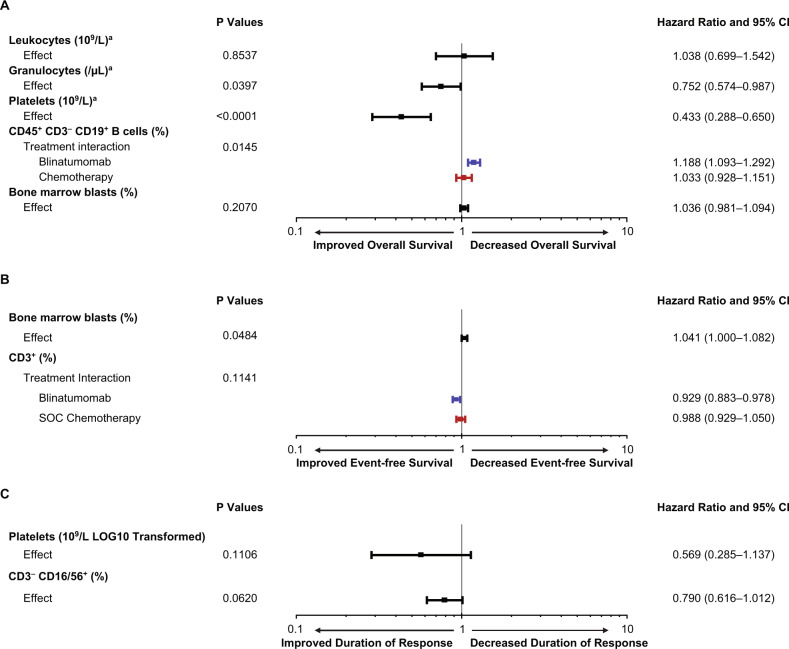
Baseline biomarkers evaluated for association with overall survival, event-free survival, and duration of response. **a** Overall survival. **b** Event-free survival. **c** Duration of response. For overall survival, a hazard ratio of <1 indicates that higher levels of the biomarker were prognostic or predictive for improved overall survival. ^a^LOG10 transformed. CD45^+^ CD3^−^ CD19^+^ B cells are expressed as a percentage of circulating lymphocytes. Bone marrow blasts are a percentage of all nucleated marrow cells. CI confidence interval.

**Fig. 2:**
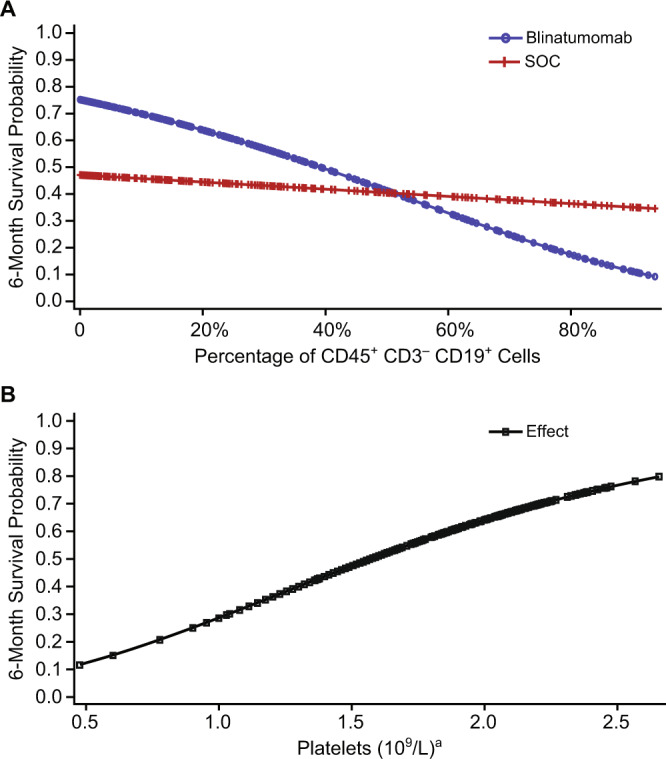
Baseline biomarkers and 6-month survival probabilities. **a** A lower percentage of CD45^+^ CD3^−^ CD19^+^ B lymphocytes at baseline was associated with higher 6-month survival probability in the blinatumomab group; this association was less pronounced in the chemotherapy (SOC) group. **b** For both treatment groups combined, higher platelet counts at baseline were associated with higher 6-month survival probability. ^a^LOG10 transformed.

We first sought to correlate baseline disease burden, bone marrow function, and effector T-cell numbers with survival outcome. A Cox regression analysis for overall survival was performed (Fig. 1a), where lower HR represented improved survival. No relationship was shown between morphologically determined bone marrow blast percentage and survival (HR, 1.04; 95% CI, 0.98–1.09). In contrast, a higher proportion of CD45^+^ CD3^–^ CD19^+^ B cells at baseline was predictive and associated with inferior overall survival in the blinatumomab group (HR, 1.19; 95% CI, 1.09–1.29) but not in the chemotherapy group (HR, 1.03; 95% CI, 0.93–1.15) (Fig. 2a). In terms of baseline bone marrow function, a higher platelet (HR, 0.43; 95% CI, 0.29–0.65) or granulocytes (HR, 0.75; 95% CI, 0.57–0.99) was prognostic for improved overall survival. For each 1-log increase in platelets at baseline, 6-month survival probability increased by ~30% (Fig. 2b). Preliminary univariate analyses for all biomarkers are included in Supplementary Materials Table S1.

### Event-free survival

No strong predictive or prognostic biomarkers were identified for event-free survival in the multivariate analysis (Fig. 1b). In the multivariate analysis for improved event-free survival, the HR for lower percentage of baseline bone marrow blasts was 1.04 (95% CI, 1.0–1.08). The HR for higher percentage of CD3^+^ T cells at baseline was 0.93 (95% CI, 0.88–0.98) in the blinatumomab group and 0.99 (95% CI, 0.93–1.05) in the chemotherapy group. Univariate analyses for all biomarkers are included in Supplementary Materials Table S2.

### Duration of response

No predictive or prognostic biomarkers were identified for duration of response in the multivariate analysis (Fig. 1c). Univariate analyses from the screening phase for all biomarkers are included in Supplementary Materials Table S3.

### Hematologic remission

**Fig. 3:**
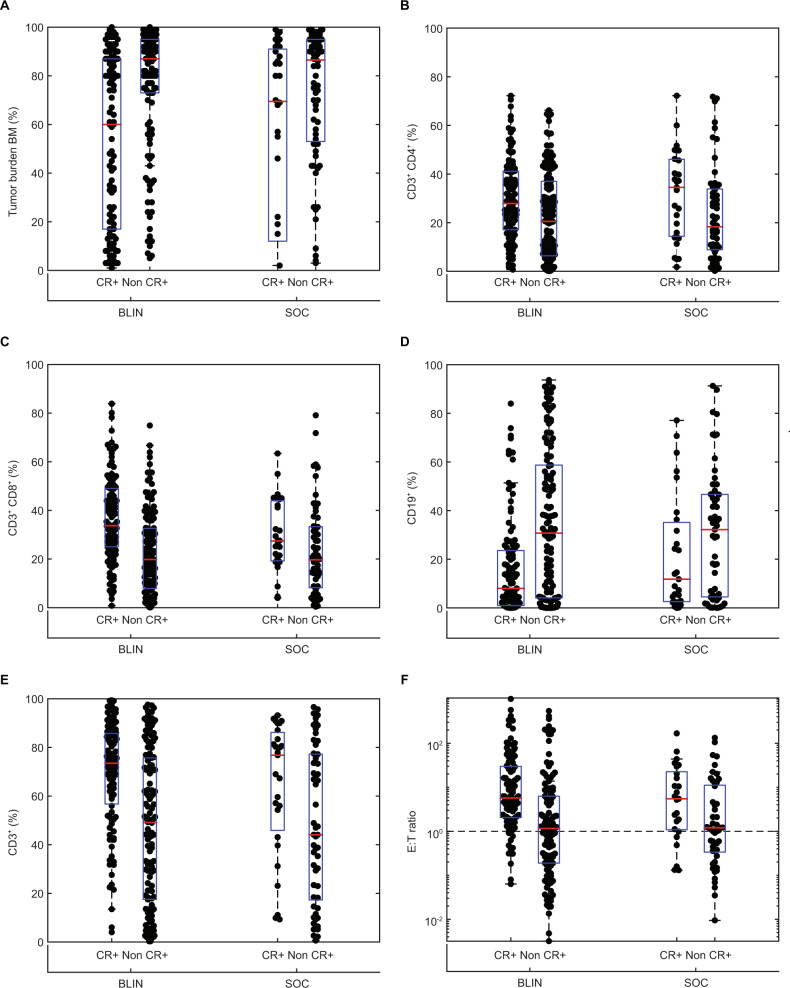
Relationship between baseline biomarkers and hematologic remission during blinatumomab (BLIN) or chemotherapy (SOC). Percentage of (**a**) bone marrow (BM) blasts, (**b**) CD3^+^ CD4^+^ T cells, (**c**) CD3^+^ CD8^+^ T cells, (**d**) CD19^+^ B cells, and (**e**) CD3^+^ T cells at baseline in patients with (CR+) or without (Non CR+) hematologic remission (CR, CRh, and CRi). **f** Baseline CD3^+^:CD19^+^ (E:T) ratio in patients with or without hematologic remission. The red horizontal line indicates the median value and the box includes the 25th and 75th percentile values. *CR* complete remission with full hematologic recovery, *CRh* complete remission with partial hematologic recovery, *CRi* complete remission with incomplete hematologic recovery, *SOC* standard of care.

In the primary analysis of TOWER, the hematologic remission rates for blinatumomab and chemotherapy were 43.9% vs. 24.6%, respectively (*P* < 0.001), with CR in 33.6% vs. 15.7%, CRh in 8.9% vs. 4.5%, and CRi in 1.5% vs. 4.5% of patients [6]. In a univariate analysis of baseline biomarkers, patients who achieved hematologic remission with blinatumomab or chemotherapy compared with those who did not, had lower baseline bone marrow disease burden and a higher percentages of total T cells, T helper and T suppressor cells, characterized by CD45^+^ CD3^+^ T cells, CD45^+^ CD3^+^ CD4^+^ T cells, and CD45^+^ CD3^+^ CD8^+^ T cells, respectively (Fig. 3). In both treatment groups, the baseline E:T ratio was higher in patients achieving hematologic remission compared with patients without hematologic remission.

**Fig. 4:**
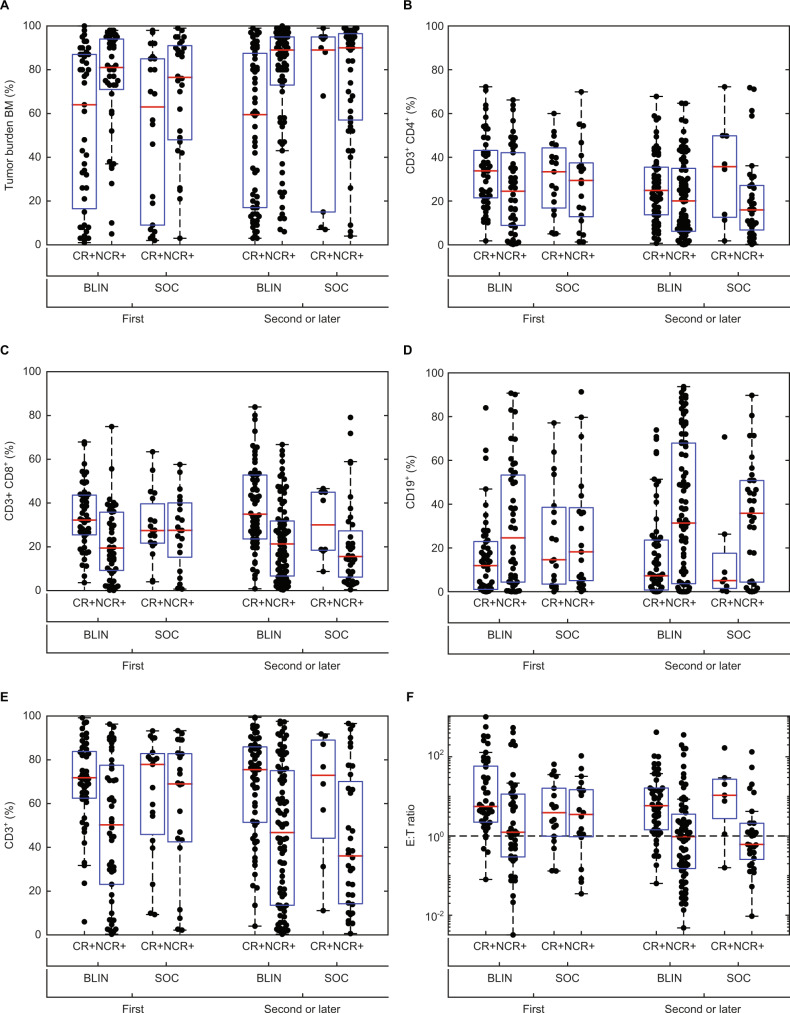
Relationship between baseline biomarkers and hematologic remission during blinatumomab (BLIN) or chemotherapy (SOC) as first salvage therapy or as second or later salvage therapy. Percentage of (**a**) bone marrow (BM) blasts, (**b**) CD3^+^ CD4^+^ T cells, (**c**) CD3^+^ CD8^+^ T cells, (**d**) CD19^+^ B cells, and (**e**) CD3^+^ T cells at baseline in patients with (CR+) or without (Non CR+) hematologic remission (CR, CRh, and CRi). **f** Baseline CD3^+^:CD19^+^ (E:T) ratio in patients with or without hematologic remission. The red horizontal line indicates the median value and the box includes the 25th and 75th percentile values. CR complete remission with full hematologic recovery, CRh complete remission with partial hematologic recovery, CRi complete remission with incomplete hematologic recovery, SOC standard of care.

As increased number of prior lines of therapy negatively influence BCP-ALL outcomes [17, 18], we looked for differences in the tumor burden or immune status of patients treated in first vs. second or later salvage therapy. Exploratory analysis suggests lower baseline bone marrow blasts and CD19^+^ cells were associated with higher hematologic remission rate for both blinatumomab and chemotherapy groups in first salvage as well as in second or later salvage (Fig. 4). In contrast, the association between immune profile and hematologic remission differed between blinatumomab and chemotherapy for treatment in first salvage. Patients treated with blinatumomab had a higher hematologic remission rate at first salvage if there was a higher proportion of CD3^+^ CD4^+^ T cells or CD3^+^ CD8^+^ T cells, as well as a higher E:T ratio at baseline, whereas this association was weaker for patients treated with chemotherapy. Therefore, immune status appeared to be predictive of response to blinatumomab for patients treated at first salvage.

**Fig. 5:**
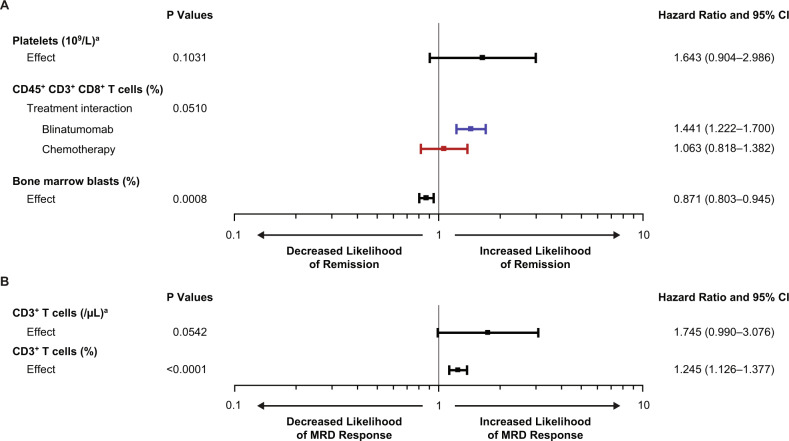
Baseline biomarkers evaluated for association with hematologic remission and minimal residual disease. **a** Hematologic remission. **b** minimal residual disease (MRD) response. When the odds ratio was >1, the biomarker was prognostic or predictive for increased likelihood of hematologic remission or MRD response. CR/CRh/CRi = complete remission with full (CR), partial (CRh), or incomplete (CRi) recovery of peripheral blood counts. MRD response = an MRD level below 10^−4^ (by PCR or flow cytometry). ^a^LOG10 transformed. T cells are expressed as a percentage of circulating lymphocytes. Bone marrow blasts are a percentage of all nucleated marrow cells. CI confidence interval, PCR polymerase chain reaction.

In the multivariate regression analysis (Fig. 5a), a lower percentage of bone marrow blasts at baseline (OR, 0.87; 95% CI, 0.80–0.95) was prognostic for hematologic remission, and platelet count at baseline was not prognostic (OR, 1.64; 95% CI, 0.90–2.99). A higher CD45^+^ CD3^+^ CD8^+^ T-cell percentage at baseline was predictive and associated with hematologic remission in the blinatumomab group (OR, 1.44; 95% CI, 1.22–1.70) but not in the chemotherapy group (OR, 1.06; 95% CI, 0.82–1.38). Absolute numbers of T-cells at baseline were not found to correlate with response to blinatumomab (Table S4). Preliminary univariate analyses in the screening phase for all biomarkers are included in Supplementary Materials Table S4.

### MRD response

In a multivariate regression analysis for determinants of MRD response (Fig. 5b), a higher percentage of CD3^+^ T cells in blood at baseline was prognostic for MRD response (OR, 1.25; 95% CI, 1.13–1.38). Univariate analyses in the screening phase for all biomarkers are included in Supplementary Materials Table S5.

### Adverse events

In the univariate logistic regression analysis for adverse events of grade ≥3 neurologic events, grade ≥3 infections, or grade ≥3 cytokine release syndrome, none of the examined biomarkers were significant for either treatment group, including percentage of bone marrow blasts, blast count, absolute count of CD45^+^ CD3^+^ CD8^+^ cells, percentage of CD45^+^ CD3^+^ CD8^+^ cells, absolute count of CD3^+^ cells, percentage of CD3^+^ cells, absolute count of CD3^−^ CD19^+^cells and percentage of CD3^−^ CD19^+^ cells (Table 2). The results of each biomarker in this analysis are summarized in Table 3.

**Table 2:** Odds ratios for adverse events of interest by baseline biomarker.

Adverse Event of Interest	Blinatumomab	Chemotherapy
Biomarker	Odds Ratio (95% CI)	Odds Ratio (95% CI)
Grade ≥3 neurologic events		
Bone marrow blast (%)	1.00 (0.99–1.02)	1.00 (0.99–1.02)
Blast count (10^9^/L)	0.97 (0.90–1.04)	0.96 (0.84–1.09)
CD45^+^ CD3^+^ CD8^+^ cells (%)	0.99 (0.97–1.02)	1.00 (0.97–1.03)
CD45^+^ CD3^+^ CD8^+^ cell counts (/µL)	1.00 (1.00–1.00)	1.00 (1.00–1.00)
CD3^+^ (%)	0.99 (0.98–1.01)	1.00 (0.98–1.01)
CD3^+^ cell counts (/µL)	1.00 (1.00–1.00)	1.00 (1.00–1.00)
CD3^−^CD19^+^ (%)	1.01 (1.00–1.03)	0.99 (0.96–1.02)
CD3^−^CD19^+^ cell counts (/µL)	1.00 (1.00–1.00)	1.00 (1.00–1.00)
Grade ≥3 infections		
Bone marrow blast (%)	1.00 (0.99–1.01)	1.01 (1.00–1.02)
Blast count (10^9^/L)	0.99 (0.98–1.01)	1.01 (0.98–1.05)
CD45^+^ CD3^+^ CD8^+^ cells (%)	0.99 (0.97–1.00)	1.01 (0.99–1.03)
CD45^+^ CD3^+^ CD8^+^ cell counts (/µL)	1.00 (1.00–1.00)	1.00 (1.00–1.00)
CD3^+^ (%)	0.99 (0.98–1.00)	1.00 (0.99–1.01)
CD3^+^ cell counts (/µL)	1.00 (1.00–1.00)	1.00 (1.00–1.00)
CD3^−^ CD19^+^ (%)	1.00 (1.00–1.01)	1.02 (1.00–1.03)
CD3^−^ CD19^+^ cell counts (/µL)	1.00 (1.00–1.00)	1.00 (1.00–1.00)
Grade ≥3 cytokine release syndrome		
Bone marrow blast (%)	1.02 (1.00–1.05)	–
Blast count (10^9^/L)	0.99 (0.93–1.05)	–
CD45^+^ CD3^+^ CD8^+^ cells (%)	1.02 (1.00–1.05)	–
CD45^+^ CD3^+^ CD8^+^ cell counts (/µL)	1.00 (1.00–1.00)	–
CD3^+^ (%)	1.03 (1.00–1.05)	–
CD3^+^ cell counts (/µL)	1.00 (1.00–1.00)	–
CD3^−^ CD19^+^ (%)	0.99 (0.96–1.01)	–
CD3^−^ CD19^+^ cell counts (/µL)	1.00 (1.00–1.00)	–

T cells were measured as a percentage of all lymphocytes in the blood.

*CI* confidence interval.

**Table 3:** Summary of prognostic (both treatment groups) or predictive (either treatment groups).

Outcome	Baseline biomarker	Finding
Improved overall survival	Higher neutrophil count	Prognostic
	Higher platelet count	Prognostic
	Lower percentage of CD45^+^ CD3^–^ CD19^+^ cells	Predictive (blinatumomab)
Increased likelihood of CR/CRh/CRi	Lower percentage of bone marrow blasts	Prognostic
	Higher percentage of CD45^+^ CD3^+^ CD8^+^ T cells in first salvage	Predictive (blinatumomab)
Increased likelihood of MRD response	Higher percentage of CD3^+^ T cell cells	Prognostic
Adverse events of interest	Percentage of bone marrow blasts	None
	Percentage of CD45^+^ CD3^+^ CD8^+^ cells	None
	Percentage of CD3^+^ cells	None

*CR* complete remission with full hematologic recovery, *CRh* complete remission with partial hematologic recovery, *CRi* complete remission with incomplete hematologic recovery, *MRD* minimal residual disease.

## Discussion

In this biomarker analysis, clinically relevant baseline laboratory markers associated with survival outcome after blinatumomab or chemotherapy in patients with BCP-ALL were examined. We found that higher granulocytes and platelet counts were prognostic for improved overall survival in either treatment group. The strongest predictive marker for improved overall survival after blinatumomab was a lower percentage of peripheral blood CD45^+^ CD3^−^ CD19^+^ B lymphocytes. No strong predictive or prognostic markers for event-free survival and duration of response were identified.

For biomarkers of hematologic remission, a multivariate analysis revealed that a lower percentage of bone marrow blasts at baseline was prognostic in either treatment group. This is consistent with the notion that treating patients with lower disease burden may result in a higher treatment response. Thus, confirming that this hypothesis applies to patients treated with either immunotherapy or chemotherapy. Furthermore, higher baseline platelet count was also prognostic for response to therapy, suggesting this biomarker may function as a surrogate of preserved bone marrow function. Consistent with the blinatumomab mode of action, a higher baseline percentage of CD3^+^ CD8^+^ effector T cells was a predictive marker of response to blinatumomab but not chemotherapy. Although blinatumomab can eradicate tumors at low effector-to-target (E:T) cell ratios through serial lysis [7, 19, 20], greater percentages of CD3^+^ T cell with correspondingly lower percentages of CD19^+^ B cells (i.e., lower tumor burden) create the optimal tumor-killing environment [8]. In this and other studies, absolute numbers of T-cells at baseline were not found to correlate with response to blinatumomab [5, 7, 10].

In a single-arm, phase 2 study of adults with relapsed or refractory BCP-ALL, 43 of 59 (73%) patients with <50% blasts at baseline and 38 of 130 (29%) patients with ≥50% blasts at baseline achieved hematologic remission [8]. In the randomized, phase 3 TOWER study, the OR for hematologic remission with blinatumomab compared with chemotherapy was 3.65 (95% CI, 1.63–8.17) in patients with <50% blasts at baseline and 1.99 (95% CI, 1.12–3.55) in patients with ≥50% blasts at baseline [6]. In the original TOWER study, overall survival was generally consistent across subgroups, including blasts at baseline. These previous results, combined with the findings from the multivariate models of this biomarker analysis, suggest that cytoreduction or debulking treatment to reduce blast counts before initiating blinatumomab in adults with relapsed or refractory BCP-ALL could increase hematologic remission rates but may not have a significant effect on overall survival.

Other results from this study suggested that if another treatment is used before an immunotherapeutic such as blinatumomab, then a prior treatment that maintains the number and fitness of T cells would be preferred. A higher percentage of CD45^+^ CD3^+^ CD8^+^ T-cells at baseline was predictive for hematologic remission in the blinatumomab group of this study. The baseline CD45^+^ CD3^+^ CD8^+^ T-cell percentage was not predictive for hematologic remission in the chemotherapy group, suggesting that the number of T cells at baseline might be an important biomarker for response to immunotherapy-based drugs such as blinatumomab, but not chemotherapy. Zhu et al. [14] also demonstrated that patients who had a higher percentage of T cells and a lower percentage of B cells at baseline responded better to blinatumomab. In another study, patients who responded to blinatumomab had more pronounced T-cell expansion, which was associated with proliferation of CD4^+^ and CD8^+^ T cells and memory subsets [7].

In this biomarker analysis, higher CD3^+^ T-cell percentage were prognostic for a higher likelihood of MRD negativity in either treatment group, with no predictive factor for differential MRD response between treatment groups identified. These findings are corroborated by Zugmaier et al. [12], who showed that long-term survivors (≥30 months) were MRD negative and had greater CD3^+^ T-cell expansion.

This biomarker analysis did not identify a prognostic or predictive factor for severe (grade ≥ 3) neurologic events, infections, or cytokine release syndrome. Thus, no biomarker for blinatumomab adverse events was identified. In contrast, these results are different compared with other publications in patients undergoing CD19 chimeric antigen receptor (CAR) T-cell therapy where serum cytokines were found to be associated with neurotoxicity [21, 22]. However, these discrepancies can be explained by the different platforms and methods used to measure cytokines, including differences in sensitivity and timepoints for cytokine analysis, as well as the high number of CAR T-cells reinfused.

It should be noted that this descriptive analysis of biomarkers should be considered exploratory, and it was not considered in the original study design or sample size calculations for the TOWER clinical trial. Dexamethasone premedication in a selected subgroup of patients may have altered the number and function of T cells after baseline levels were assessed. Thus, the observed associations between baseline blast count and hematologic remission with blinatumomab or chemotherapy may have been influenced by the intervening use of dexamethasone. Lastly, measuring plasma cytokine concentrations may be warranted in future studies.

In conclusion, this analysis of a large, phase 3 comparison of blinatumomab or chemotherapy for salvage therapy in adults with relapsed or refractory BCP-ALL identified baseline biomarkers that may be prognostic or predictive for clinical outcomes, including overall survival, hematologic remission, and MRD response. No examined biomarker was significant for adverse events. Although these studies have identified potential prognostic and predictive associations with blinatumomab therapy, validation in a prospective study is needed to confirm patient subgroups most likely to benefit from blinatumomab.

## Supplementary information

Supplemental Materials
