# Supplementary material for: Biomarkers associated with blinatumomab outcomes in acute lymphoblastic leukemia
Source: Leukemia. 2021 Feb 4;35(8):2220–31. doi: 10.1038/s41375-020-01089-x (PMC8324476; doi:10.1038/s41375-020-01089-x)
Supplement: Supplementary file 1 — Supplemental Materials [file 41375_2020_1089_MOESM1_ESM.docx]

# Supplemental Material

## Table S1: Overall Survival

| Biomarker | Parameter Estimate | Standard Error | P value |
| --- | --- | --- | --- |
| Leukocytes (10^^9^/L) |  |  |  |
| Effect | -0.60314 | 0.26058 | 0.0206 |
| Treatment Interaction | 0.53428 | 0.30518 | 0.0800 |
| Lymphocytes (10^^9^/L) |  |  |  |
| Effect | -0.25228 | 0.25005 | 0.3130 |
| Treatment Interaction | -0.04325 | 0.31261 | 0.8900 |
| Granulocytes (/µL) |  |  |  |
| Effect | -0.58795 | 0.15130 | 0.0001 |
| Treatment Interaction | 0.13344 | 0.17825 | 0.4541 |
| Platelets (10^^9^/L) |  |  |  |
| Effect | -1.29249 | 0.26754 | <0.0001 |
| Treatment Interaction | 0.09236 | 0.32845 | 0.7786 |
| Neutrophils (10^^9^/L) |  |  |  |
| Effect | -0.54052 | 0.16207 | 0.0009 |
| Treatment Interaction | 0.21929 | 0.19125 | 0.2516 |
| CD45+ CD3+ CD4+ T cells (/µL) |  |  |  |
| Effect | -0.62200 | 0.24957 | 0.0128 |
| Treatment Interaction | -0.02024 | 0.28382 | 0.9431 |
| CD45+ CD3+ CD4+ T cells (%) |  |  |  |
| Effect | -0.01192 | 0.00714 | 0.0951 |
| Treatment Interaction | -0.01361 | 0.00877 | 0.1208 |
| CD45+ CD3+ CD8+ cells (/µL) |  |  |  |
| Effect | -0.48229 | 0.24189 | 0.0462 |
| Treatment Interaction | -0.10218 | 0.28531 | 0.7203 |
| CD45+ CD3+ CD8+ cells (%) |  |  |  |
| Effect | -0.00471 | 0.00801 | 0.5561 |
| Treatment Interaction | -0.01725 | 0.00951 | 0.0700 |
| CD45+ CD3- CD19+ (%)* |  |  |  |
| Effect | 0.06324 | 0.05001 | 0.2062 |
| Treatment Interaction | 0.12471 | 0.05702 | 0.0288 |
| CD3- CD16+ CD56+ (/µL) |  |  |  |
| Effect | -0.31765 | 0.23737 | 0.1810 |
| Treatment Interaction | 0.24750 | 0.28675 | 0.3881 |
| CD3- CD16+ CD56+ (%) |  |  |  |
| Effect | 0.00074 | 0.01416 | 0.9585 |
| Treatment Interaction | -0.01283 | 0.01674 | 0.4433 |
| Bone marrow blasts (%)* |  |  |  |
| Effect | 0.11712 | 0.03936 | 0.0029 |
| Treatment Interaction | 0.02019 | 0.04868 | 0.6783 |
| CD3+ (/µL) |  |  |  |
| Effect | -0.57232 | 0.25359 | 0.0241 |
| Treatment Interaction | -0.11350 | 0.29158 | 0.6971 |
| CD3+ (%) |  |  |  |
| Effect | -0.00630 | 0.00414 | 0.1281 |
| Treatment Interaction | -0.01227 | 0.00499 | 0.0139 |

Treatment interaction: Reference group is standard of care chemotherapy

*CD45+ CD3- CD19+ (%) and Bone marrow blasts (%) results have been divided by 10.

## Table S2: Event-Free Survival

| Biomarker | Parameter Estimate | Standard Error | P value |
| --- | --- | --- | --- |
| Leukocytes (10^^9^/L) |  |  |  |
| Effect | -0.03407 | 0.17507 | 0.8457 |
| Treatment Interaction | 0.15198 | 0.21577 | 0.4812 |
| Lymphocytes (10^^9^/L) |  |  |  |
| Effect | -0.01518 | 0.17724 | 0.9317 |
| Treatment Interaction | 0.08112 | 0.23314 | 0.7279 |
| Granulocytes (/µL) |  |  |  |
| Effect | -0.10808 | 0.11627 | 0.3526 |
| Treatment Interaction | -0.02539 | 0.14367 | 0.8598 |
| Platelets (10^^9^/L) |  |  |  |
| Effect | -0.37123 | 0.22074 | 0.0926 |
| Treatment Interaction | 0.07593 | 0.27193 | 0.7801 |
| Neutrophils (10^^9^/L) |  |  |  |
| Effect | -0.10230 | 0.13549 | 0.4502 |
| Treatment Interaction | 0.01455 | 0.16251 | 0.9287 |
| CD45+ CD3+ CD4+ T cells (/µL) |  |  |  |
| Effect | -0.10328 | 0.16172 | 0.5231 |
| Treatment Interaction | 0.04032 | 0.20659 | 0.8453 |
| CD45+ CD3+ CD4+ T cells (%) |  |  |  |
| Effect | -0.00410 | 0.00506 | 0.4179 |
| Treatment Interaction | -0.00479 | 0.00658 | 0.4666 |
| CD45+ CD3+ CD8+ cells (/µL) |  |  |  |
| Effect | -0.09359 | 0.16856 | 0.5787 |
| Treatment Interaction | -0.03154 | 0.21025 | 0.8807 |
| CD45+ CD3+ CD8+ cells (%) |  |  |  |
| Effect | -0.00250 | 0.00542 | 0.6445 |
| Treatment Interaction | -0.01143 | 0.00672 | 0.0891 |
| CD45+ CD3- CD19+ (%) |  |  |  |
| Effect | 0.01969 | 0.03538 | 0.5777 |
| Treatment Interaction | 0.06888 | 0.04326 | 0.1113 |
| CD3- CD16+ CD56+ (/µL) |  |  |  |
| Effect | -0.00192 | 0.16385 | 0.9906 |
| Treatment Interaction | 0.03399 | 0.20946 | 0.8711 |
| CD3- CD16+ CD56+ (%) |  |  |  |
| Effect | 0.00309 | 0.00961 | 0.7477 |
| Treatment Interaction | -0.01568 | 0.01209 | 0.1946 |
| Bone marrow blasts (%) |  |  |  |
| Effect | 0.00309 | 0.00961 | 0.7477 |
| Treatment Interaction | -0.01568 | 0.01209 | 0.1946 |
| CD3+ (/µL) |  |  |  |
| Effect | -0.10732 | 0.17432 | 0.5381 |
| Treatment Interaction | -0.00565 | 0.21872 | 0.9794 |
| CD3+ (%) |  |  |  |
| Effect | -0.00267 | 0.00306 | 0.3817 |
| Treatment Interaction | -0.00623 | 0.00386 | 0.1065 |

## Table S3: Duration of response for patients who achieved CR/CRh*/CRi within 12 weeks of treatment initiation

| Biomarker | Parameter Estimate | Standard Error | P value |
| --- | --- | --- | --- |
| Leukocytes (10^^9^/L) |  |  |  |
| Effect | -0.31033 | 0.68945 | 0.6526 |
| Treatment Interaction | 0.57307 | 0.75631 | 0.4486 |
| Monocytes (10^^9^/L) |  |  |  |
| Effect | -0.16944 | 0.42333 | 0.6890 |
| Treatment Interaction | 0.29872 | 0.49133 | 0.5432 |
| Lymphocytes (10^9/L LOG10 Transformed) |  |  |  |
| Effect | -0.76934 | 0.74422 | 0.3013 |
| Treatment Interaction | 1.15558 | 0.84069 | 0.1693 |
| Granulocytes (/µL LOG10 Transformed) |  |  |  |
| Effect | -0.45200 | 0.56651 | 0.4250 |
| Treatment Interaction | 0.38229 | 0.60953 | 0.5306 |
| Platelets (10^^9^/L) |  |  |  |
| Effect | -3.15796 | 0.90076 | 0.0005 |
| Treatment Interaction | 3.28849 | 0.93987 | 0.0005 |
| Neutrophils (10^^9^/L) |  |  |  |
| Effect | -0.59277 | 0.58114 | 0.3078 |
| Treatment Interaction | 0.67447 | 0.62001 | 0.2767 |
| CD45+ CD3+ CD4+ T cells (/µL) |  |  |  |
| Effect | -0.76565 | 0.78812 | 0.3315 |
| Treatment Interaction | 0.75765 | 0.85634 | 0.3764 |
| CD45+ CD3+ CD4+ T cells (%) |  |  |  |
| Effect | -0.01082 | 0.01402 | 0.4402 |
| Treatment Interaction | -0.00128 | 0.01667 | 0.9386 |
| CD45+ CD3+ CD8+ cells (/µL) |  |  |  |
| Effect | -0.29161 | 0.72794 | 0.6887 |
| Treatment Interaction | 0.47098 | 0.79490 | 0.5535 |
| CD45+ CD3+ CD8+ cells (%) |  |  |  |
| Effect | -0.00359 | 0.01985 | 0.8563 |
| Treatment Interaction | 0.00126 | 0.02137 | 0.9531 |
| CD45+ CD3- CD19+ (%)* |  |  |  |
| Effect | 0.05052 | 0.10750 | 0.6384 |
| Treatment Interaction | 0.04237 | 0.12627 | 0.7372 |
| CD3- CD16+ CD56+ (/µL) |  |  |  |
| Effect | 0.01245 | 0.73243 | 0.9864 |
| Treatment Interaction | -0.32510 | 0.79141 | 0.6813 |
| CD3- CD16+ CD56+ (%) |  |  |  |
| Effect | 0.04105 | 0.04792 | 0.3920 |
| Treatment Interaction | -0.06692 | 0.04985 | 0.1799 |
| Bone marrow blasts (%)* |  |  |  |
| Effect | 0.09997 | 0.07483 | 0.1816 |
| Treatment Interaction | -0.12577 | 0.08463 | 0.1373 |
| CD3+ (/µL) |  |  |  |
| Effect | -0.70571 | 0.83768 | 0.3996 |
| Treatment Interaction | 0.90618 | 0.91156 | 0.3202 |
| CD3+ (%) |  |  |  |
| Effect | -0.00840 | 0.00955 | 0.3791 |
| Treatment Interaction | 0.00356 | 0.01115 | 0.7492 |
| CD3%/Bone marrow blast% ratio |  |  |  |
| Effect | -0.03346 | 0.04454 | 0.4526 |
| Treatment Interaction | 0.00839 | 0.04944 | 0.8653 |
| CD3%/Bone marrow blast% ratio (<8 vs >= 8) |  |  |  |
| Effect | -0.36667 | 0.65708 | 0.5769 |
| Treatment Interaction | -0.17625 | 0.78943 | 0.8234 |

## Table S4: Hematologic Remission (CR/CRh*/CRi) Within 12 Weeks of Treatment Initiation

| Biomarker | Parameter Estimate | Standard Error | P value |
| --- | --- | --- | --- |
| Leukocytes (10^^9^/L) |  |  |  |
| Effect | 0.09336 | 0.39752 | 0.8143 |
| Treatment Interaction | -0.30402 | 0.45040 | 0.4997 |
| Lymphocytes (10^^9^/L) |  |  |  |
| Effect | -0.09453 | 0.41777 | 0.8210 |
| Treatment Interaction | 0.02796 | 0.48909 | 0.9544 |
| Granulocytes (/µL) |  |  |  |
| Effect | 0.46302 | 0.29857 | 0.1210 |
| Treatment Interaction | -0.05875 | 0.34196 | 0.8636 |
| Platelets (10^^9^/L) |  |  |  |
| Effect | 0.62963 | 0.47368 | 0.1838 |
| Treatment Interaction | 0.54500 | 0.56741 | 0.3368 |
| Neutrophils (10^^9^/L) |  |  |  |
| Effect | 0.35953 | 0.32995 | 0.2759 |
| Treatment Interaction | -0.03957 | 0.37222 | 0.9153 |
| CD45+ CD3+ CD4+ T cells (/µL) |  |  |  |
| Effect | 0.43168 | 0.43427 | 0.3202 |
| Treatment Interaction | -0.27043 | 0.49122 | 0.5820 |
| CD45+ CD3+ CD4+ T cells (%) |  |  |  |
| Effect | 0.01490 | 0.01164 | 0.2007 |
| Treatment Interaction | 0.00298 | 0.01379 | 0.8289 |
| CD45+ CD3+ CD8+ cells (/µL) |  |  |  |
| Effect | 0.49409 | 0.42913 | 0.2496 |
| Treatment Interaction | -0.04499 | 0.48943 | 0.9268 |
| CD45+ CD3+ CD8+ cells (%) |  |  |  |
| Effect | 0.12641 | 0.12177 | 0.2993 |
| Treatment Interaction | 0.30591 | 0.14591 | 0.0361 |
| CD45+ CD3- CD19+ (%)* |  |  |  |
| Effect | -0.00944 | 0.00868 | 0.2769 |
| Treatment Interaction | -0.01701 | 0.01036 | 0.1008 |
| CD3- CD16+ CD56+ (/µL) |  |  |  |
| Effect | -0.06017 | 0.38155 | 0.8747 |
| Treatment Interaction | -0.18144 | 0.43888 | 0.6793 |
| CD3- CD16+ CD56+ (%) |  |  |  |
| Effect | -0.01440 | 0.02688 | 0.5922 |
| Treatment Interaction | 0.03210 | 0.02953 | 0.2772 |
| Bone marrow blasts (%)* |  |  |  |
| Effect | -0.11083 | 0.06239 | 0.0756 |
| Treatment Interaction | -0.12930 | 0.07709 | 0.0935 |
| CD3+ (/µL) |  |  |  |
| Effect | 0.49992 | 0.45468 | 0.2716 |
| Treatment Interaction | -0.08456 | 0.51972 | 0.8708 |
| CD3+ (%) |  |  |  |
| Effect | 0.01124 | 0.00743 | 0.1305 |
| Treatment Interaction | 0.01586 | 0.00899 | 0.0778 |

Treatment interaction: Reference group is standard of care chemotherapy

*CD45+ CD3- CD19+ (%) and Bone marrow blasts (%) results have been divided by 10.

## Table S5: MRD within 12 weeks of Treatment Initiation

| Biomarker | Parameter Estimate | Standard Error | P value |
| --- | --- | --- | --- |
| Leukocytes (10^^9^/L) |  |  |  |
| Effect | -0.06913 | 0.47682 | 0.8847 |
| Treatment Interaction | -0.06111 | 0.52687 | 0.9077 |
| Lymphocytes (10^^9^/L) |  |  |  |
| Effect | -0.04285 | 0.50808 | 0.9328 |
| Treatment Interaction | 0.13954 | 0.57597 | 0.8086 |
| Granulocytes (/µL) |  |  |  |
| Effect | 0.32967 | 0.34485 | 0.3391 |
| Treatment Interaction | 0.04922 | 0.38974 | 0.8995 |
| Platelets (10^^9^/L) |  |  |  |
| Effect | 0.88766 | 0.58829 | 0.1313 |
| Treatment Interaction | -0.16908 | 0.66829 | 0.8003 |
| Neutrophils (10^^9^/L) |  |  |  |
| Effect | 0.26585 | 0.39011 | 0.4956 |
| Treatment Interaction | -0.02051 | 0.42993 | 0.9620 |
| CD45+ CD3+ CD4+ T cells (/µL) |  |  |  |
| Effect | 0.76599 | 0.57433 | 0.1824 |
| Treatment Interaction | -0.23634 | 0.63985 | 0.7119 |
| CD45+ CD3+ CD4+ T cells (%) |  |  |  |
| Effect | 0.02310 | 0.01377 | 0.0935 |
| Treatment Interaction | 0.00043 | 0.01575 | 0.9780 |
| CD45+ CD3+ CD8+ cells (/µL) |  |  |  |
| Effect | 0.49268 | 0.51524 | 0.3390 |
| Treatment Interaction | 0.07635 | 0.58003 | 0.8953 |
| CD45+ CD3+ CD8+ cells (%) |  |  |  |
| Effect | 0.01347 | 0.01455 | 0.3545 |
| Treatment Interaction | 0.01570 | 0.01638 | 0.3378 |
| CD45+ CD3- CD19+ (%)* |  |  |  |
| Effect | -0.01255 | 0.01108 | 0.2573 |
| Treatment Interaction | -0.00773 | 0.01243 | 0.5339 |
| CD3- CD16+ CD56+ (/µL) |  |  |  |
| Effect | -0.07555 | 0.45633 | 0.8685 |
| Treatment Interaction | -0.05737 | 0.50964 | 0.9104 |
| CD3- CD16+ CD56+ (%) |  |  |  |
| Effect | -0.03298 | 0.03535 | 0.3510 |
| Treatment Interaction | 0.03088 | 0.03777 | 0.4136 |
| Bone marrow blasts (%)* |  |  |  |
| Effect | -0.00690 | 0.00755 | 0.3610 |
| Treatment Interaction | -0.00375 | 0.00861 | 0.6628 |
| CD3+ (/µL) |  |  |  |
| Effect | 0.63217 | 0.56720 | 0.2651 |
| Treatment Interaction | 0.04895 | 0.64224 | 0.9392 |
| CD3+ (%) |  |  |  |
| Effect | 0.16762 | 0.09780 | 0.0866 |
| Treatment Interaction | 0.08359 | 0.11115 | 0.4521 |

Treatment interaction: Reference group is standard of care chemotherapy

*CD45+ CD3- CD19+ (%) and Bone marrow blasts (%) results have been divided by 10.
